# Trends in Prevalent Cases and Disability-Adjusted Life-Years of Depressive Disorders Worldwide: Findings From the Global Burden of Disease Study From 1990 to 2021

**DOI:** 10.1155/da/5553491

**Published:** 2025-04-24

**Authors:** Xiao-dan Chen, Feng Li, Hui Zuo, Feng Zhu

**Affiliations:** ^1^Pharmaceutical Department, Guangzhou Twelfth People's Hospital, Guangzhou, China; ^2^Prevention and Health Department, Guangzhou Twelfth People's Hospital, Guangzhou, China; ^3^Central Laboratory, Guangzhou Twelfth People's Hospital, Guangzhou, China

**Keywords:** depression, disability-adjusted life-year, disease burden, sociodemographic index

## Abstract

**Background:** Depression is a primary public health challenge that affects individuals of all ages. This study aims to reveal information on spatial and temporal changes in depression by describing temporal trend differences, regional differences, and gender differences.

**Materials and Methods:** Utilizing data from the Global Burden of Disease Study 2021 (GBD2021) from 1990 to 2021, we outlined the prevalence and burden of depression among 204 countries in 21 regions, including age and sex disparities, and explored the correlation between depressive burden and the sociodemographic index (SDI). The age-standardized rates of prevalence (ASPR), disability-adjusted life years (DALYs, age-standardized DALY rate, ASDR), and estimated annual percentage change (EAPC) were employed to evaluate the global burden of depression.

**Results:** Our study revealed a greater than 1.8-fold increase in prevalent cases and DALYs for global depressive disorder from 1990 to 2021. Globally, the age-standardized rates (ASRs) slightly declined, with a 1.32% decrease in the ASPR and a 1.84% decrease in the ASDR from 1990 to 2019. The main decline occurred from 2005 to 2010 (4.86% decrease in the ASPR and 6.09% decrease in the ASDR), with the majority of the contributions occurring in the low-middle-SDI and low-SDI regions. The global ASPR and ASDR experienced astonishing jumps from 2019 to 2021, resulting in increases of nearly 11% in the ASPR and 13% in the ASDR. Notably, the ASPR and ASDR of depression decreased in females but increased in males from 1990 to 2019 and reversed thereafter. From 1990 to 2021, among the 21 regions, the EAPCs in most regions were >0, with the only exceptional decline occurring in East Asia in the ASPR −0.06 [95% Cl:−0.10 to −0.03]) and ASDR −0.09 [95% Cl:−0.13 to −0.05]). Compared with those in other regions, the ASPR (0.42 [95% Cl: 0.34–0.49]) and ASDR (0.53 [95% Cl: 0.46–0.61]) were greater in high-income North America. Among the five SDI regions, the largest increases in ASPR (0.25 [95% Cl: 0.21–0.30]) and ASDR (0.31 [95% Cl: 0.26–0.37]) occurred in the high-SDI region, with the majority of the contributions occurring from 2019 to 2021. Worldwide, a decreasing trend and significant associations between the ASPR and the SDI (*R* = −0.22, *p*=0.0013) and between the ASDR and the SDI (*R* = −0.28, *p* < 0.001) were observed.

**Conclusion:** Depression remains a serious challenge worldwide. The trends in depression burden varied across regions and groups. A vibrant socioeconomic environment could have a positive impact on the disease burden. Mental health should be incorporated into public health preparedness and emergency plans in practical ways on the basis of the national conditions of each country.

## 1. Introduction

According to the GBD 2021, 332.41 million people suffer from depression, accounting for the largest proportion of disability-adjusted life years (DALYs) in the world (36.24%). Depression affects one in five people in their lifetime, leading to changes in mood, loss of interest, or pleasure [1]. Compared with the general population, people with depression and certain mental illnesses are 40%–60% more likely to experience premature death, mainly due to undiagnosed and untreated physical health issues such as diabetes, cancer, cardiovascular disease, HIV infection, and suicide [2, 3]. It is estimated that up to 60% of individuals who commit suicide have major depression [4]. While adults make up the largest group affected by depression, young people with major depression are 30 times more likely to be self-injured [5]. In the elderly population, depression ranks as the second most common psychiatric disorder [6]. The lifetime prevalence of major depressive disorder (MDD) has reached 20% [7]. The long-term course of depression has been characterized as highly recurrent, often leading to significant costs for both patients and society [8]. The total global cost of mental illness was projected to reach $16.3 trillion from 2011 to 2030 [9]. An action plan aimed at addressing mental illness states that suicide rates should decrease by 10% by 2020 and that medical services for mental illnesses, including moderate to severe depression, should increase by 20% [10].

The Global Burden of Disease (GBD) study provides detailed data on various diseases in 204 countries in 21 regions globally. The GBD database offers more comprehensive information on the global prevalence of depression and classifies depression into two main categories: MDD and dysthymia. Using depression data from 1990 to 2021 in the GBD database, we examined temporal trends and spatial, sex, and age distributions of depression and correlations with the sociodemographic index (SDI).

We present the prevalence and DALY rates and their estimated annual percentage changes (EAPCs) and attempt to explain the sudden drop in the disease burden in 2005, which has never been described in previous studies, and we further compared the trends in the depression burden between 1990–2019 and 2019–2021 in different regions. Our findings complement the details of the global burden puzzle for depression and hold significant scientific value for the advancement of neuroscience, psychology, and sociology.

## 2. Methods

### 2.1. Data Sources

The data were acquired from the GBD Results Tool (http://ghdx.healthdata.org/gbd-results-tool). GBD2021 provided the latest and most comprehensive epidemiological data on 371 diseases and injuries worldwide from 1990 to 2021 [11]. For this study, we extracted depression data for three age groups (<20, 20–54, and 55+) and sex data from 1990 to 2021 in 21 GBD regions and 204 countries.

### 2.2. Classification and Definitions

#### 2.2.1. Depressive Disorders

In GBD 2021, depression includes MDD and dysthymia. The diagnostic criteria for MDD are the DSM-IV-TR (296.21–24, 296.31−34) and the ICD-10 (F32.0–9, F33.0–9). The diagnostic criteria of the DSM-IV-TR (300.4) and the ICD-10 (F34.1) are used to assess dysthymia.

#### 2.2.2. SDI

The SDI is a comprehensive indicator of a country's per capita lag-distributed income, average education, and total fertility rate for women under 25 years of age [12]. Like GBD 2019, the GBD 2021 database also divides countries and regions around the world into five SDI regions. All values were divided into appropriate groups, and each indicator was expressed as an uncertainty interval (UI) via the 25th and 975th ordered 1000 plotted values of the posterior distribution [13].

#### 2.2.3. ASR

The age-standardized rate (ASR) is often used to eliminate the differential effects of different age structures among groups, making them comparable. The age-standardized rates of prevalence (ASPR) reflect age-independent prevalence levels and are used to compare prevalence data across time areas or different periods. For the calculation and application of ASR, please refer to previous literature [13], and it should be noted that GBD2021 has updated age-specific population estimates [12]. We used the ASR to quantify the prevalence of depression in terms of sex, region, and DALY trends (age-standardized DALY rate, ASDR) [14].

#### 2.2.4. EAPC

EAPC is a well-established method for characterizing ASR via regression models that quantify the average annual rate of change over a specific period. For the calculation formula of the linear regression model, please refer to previous literature [15]. All the statistics were analyzed via R software (R Core Team, version 4.3.3, 2024), and a bilateral *p* value < 0.05 was considered statistically significant [14].

Temporal trends, represented by ASPR and ASDR, were analyzed via joinpoint regression (version 5.1.0.0). *Z* tests were used to evaluate hypotheses about split points. In reference to annual percentage change (APC) [16], a time series was constructed and treated as an independent variable.

### 2.3. Analytic Strategy

We analyzed changes in depression incidence and burden, including prevalence, DALYs, and corresponding ASRs, in 204 countries spanning 21 regions from 1990 to 2021. This study provides an up-to-date portrait of the depression burden worldwide, highlighting temporal and spatial trends from 1990 to 2021 [17] and particularly significant transitional changes between 1990–2019 and 2020–2021.

Initially, we modeled the global prevalence and DALYs of depression from 1990 to 2021 and then estimated the burden of depression by age, sex, country, and region. The impact of varying development levels on the disease burden was assessed via the SDI for depression [10]. The visual indicators, correlations, and coefficients between factors were analyzed via R software. Maps were created via an online visualization tool (GBD Compare|IHMEViz Hub [healthdata.org]).

## 3. Results

### 3.1. Global Burden of Depressive Disorder

**Figure 1:**
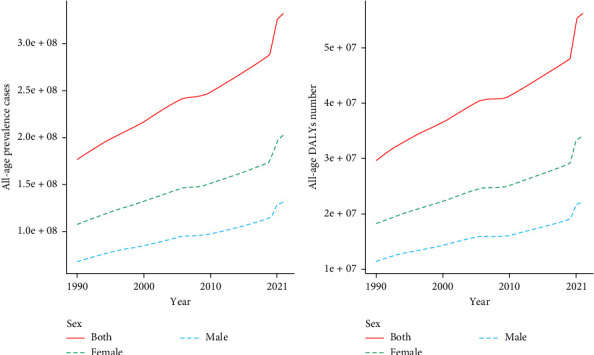
Prevalent cases and DALYs of depression in all age groups, 1990–2021. (a) Prevalence cases; (b) DALYs. DALY, disability-adjusted life-year.

Globally, the prevalence of depressive disorders increased from 1990 to 2021, with an increase of 88.52%. Compared with males, females presented a greater percentage of prevalent cases from 1990 to 2021, with 201.27 million females (95% UI: 179.87–228.46 million) and 131.14 million males (95% UI: 117.40–147.88 million) in 2021. However, males exhibited slightly greater growth (91.03%) than females did (86.92%) (Figure 1a and Table 1); similarly, the DALYs rapidly increased during the same period for both males and females. In 2021, the DALYs of females (34.12 million, 95% UI: 23.80–46.22 million) were greater than those of males (22.21 million, 95% UI: 15.50–30.32 million). Nonetheless, males experienced slightly greater growth (93.73%) than females did (87.36%) (Figure 1b and Table 1).

Globally, the ASPR increased from 3599.67 per 100,000 (95% UI: 3251.91–4023.21) in 1990–4006.82 per 100,000 (95% UI: 3581.26–4539.01) in 2021, with an ASPR-related EAPC of 0.11 (95% CI: 0.09–0.14); the ASDR also increased from 600.52 per 100,000 (95% UI: 420.94–818.45) in 1990–681.14 per 100,000 (95% UI: 475.19–923.83) in 2021, with an ASDR-related EAPC of 0.13 (95% CI: 0.11–0.16). By sex, differences were observed in the EAPC (ASPR-related EAPC of 0.12 [95% CI: 0.10–0.15] in males vs. ASPR-related EAPC of 0.11 [95% CI: 0.08–0.13] in females and ASDR-related EAPC of 0.15 [95% CI: 0.13–0.18] in males vs. ASDR-related EAPC of 0.12 [95% CI: 0.10–0.15] in females) (Table 1).

Among the 21 GBD regions, the EAPCs in most regions were >0, and the five highest ASPR- and ASDR-related EAPCs were shared by high-income North America (0.42, 95% CI: 0.34–0.49), Central Latin America (0.26, 95% CI: 0.21–0.31), Andean Latin America (0.18, 95% CI: 0.07–0.31), high-income Asia Pacific (0.17, 95% CI: 0.11–0.24), and southern sub-Saharan Africa (0.17, 95% CI: 0.11–0.25) for the ASPR; high-income North America (0.53, 95% CI: 0.46–0.61), Central Latin America (0.31, 95% CI: 0.25–0.38), Andean Latin America (0.22, 95% CI: 0.08–0.38), high-income Asia Pacific (0.22, 95% CI: 0.14–0.30), and southern sub-Saharan Africa (0.20, 95% CI: 0.13–0.29) for the ASDR. The only decrease in the EAPC occurred in East Asia, with −0.06 (95% CI: −0.10 to −0.03) for the ASPR and −0.09 (95% CI: −0.13 to −0.05) for the ASDR (Table 1).

**Figure 2:**
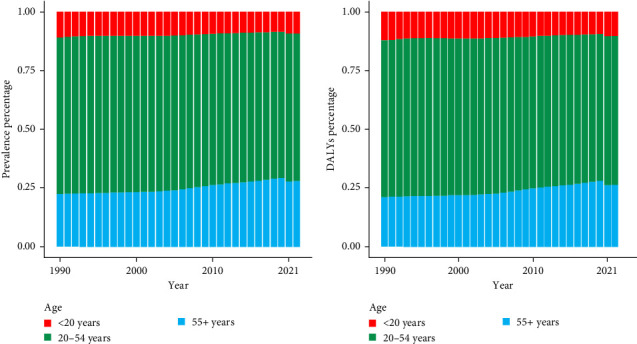
Prevalent cases and DALYs of depression in all age groups, 1990–2021. (a) Prevalent cases; (b) DALYs. DALY, disability-adjusted life-year.

Globally, the percentages of prevalent cases and DALYs vary across different age groups. The percentage of prevalent cases decreased in those aged <20 years (from 10.96% in 1990 to 8.38% in 2019). In 2020, the percentage of prevalent cases suddenly increased to 9.16%, and this percentage was maintained in 2021. For those aged 20–54 years, the percentage of prevalent cases increased slightly from 1990 to 1994 but then decreased from 1995 onwards, and this downward trend changed its momentum in 2020 and 2021, with a visible increase. For those aged 55+ years, the percentage of prevalent cases increased from 22.35% to 29.24% from 1990 to 2019 and fell below 28% in 2020 and 2021 (Figure 2a). The percentages of DALYs fluctuated from 1990 to 2021 (Figure 2b). Moreover, the most prevalent cases and DALYs were observed among people aged 20–54 years from 1990 to 2021.

**Figure 3:**
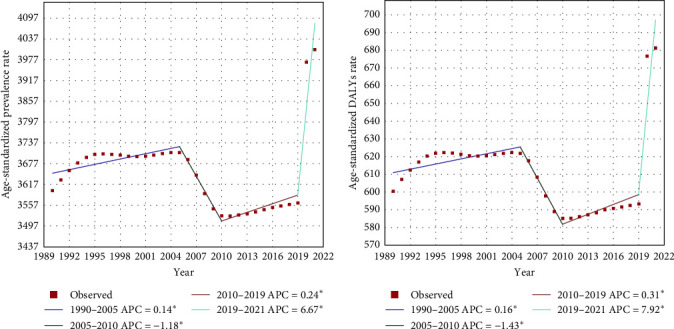
Temporal trends of depression, 1990–2021. (a) ASPRs; (b) ASDRs. *⁣*^*∗*^ Indicates that the APC is significantly different at the *α* = 0.05 level; APC, annual percentage change; ASDR, age-standardized DALY rate; ASPR, age-standardized rates of prevalence; DALY, disability-adjusted life-year.

Worldwide, there were significant changes in three turning points in 2005, 2010, and 2019 during the ASPR. Two mild stages of elevation were observed from 1990–2005 (APC = 0.14%, *p* > 0.05) and 2010–2019 (APC = 0.24%, *p* > 0.05). Between the two stages, a sharp decreasing trend was evident from 2005 [3709.09 per 100,000 persons (95% UI: 3366.48–4117.97)] to 2010 [3527.34 per 100,000 persons (95% UI: 3205.66–3893.16)] (APC = −1.18%, *p* > 0.05). There was a surprising jump in the ASPR from 2019 [3564.77 per 100,000 persons (95% UI: 3211.82–4002.90)] to 2021 [4006.82 per 100,000 persons (95% UI: 3581.26–4539.01)] (APC = 6.67%, *p* > 0.05) (Figure 3a). Similar trends were observed between the ASPR and ASDR (Figure 3b).

### 3.2. National Burden of Depressive Disorder

**Figure 4:**
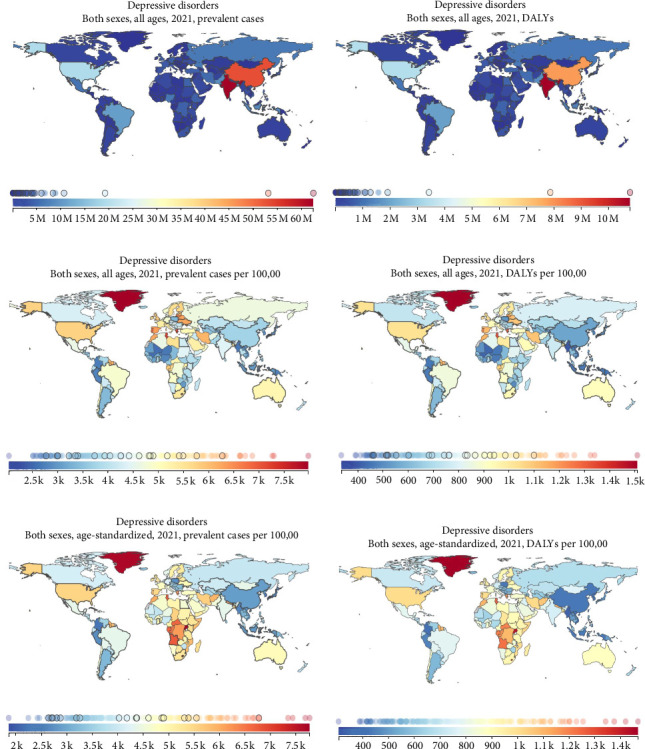
Prevalent cases, DALYs, prevalence rates, DALY rates, ASPRs, and ASDRs of depression among 204 countries and territories, 2021. (a) Prevalent cases; (b) DALYs; (c) prevalence rate; (d) DALY rate; (e) ASPRs; (f) ASDRs. Source: global burden of disease project 2021, institute for health metrics and evaluation, university of washington, seattle, WA, USA. ASPR, age-standardized rates of prevalence; ASDR, age-standardized DALY rate; DALY, disability-adjusted life-year.

Among 204 countries and regions, India had the highest number of cases (62.49 million, 95% UI: 56.02–70.50 million), followed by China, the USA, Brazil, and Pakistan in 2021. In contrast, Tokelau, Tuvalu, Marshall Islands, Dominica, and Tonga ranked among the five countries with the lowest number of cases (Figure 4a). Similarly, India had the highest DALY (8.73 million, 95% UI: 6.11–11.89 million), followed by China, the USA, Brazil, and Bangladesh in 2021 (Figure 4b).

From another perspective, Greenland had the highest prevalence rate (7979.49 per 100,000, 95% UI: 6394.22–9846.74), followed by Greece, Tunisia, Portugal, and Lithuania in 2021. In contrast, Brunei Darussalam, Singapore, Tonga Samoa, and Myanmar presented the five lowest prevalence rates in sequence (Figure 4c). Greenland had a leading DALY rate (1509.78 per 100,000, 95% UI: 999.39–2188.17); Tunisia was in the second position, with Greece, Palestine, and Portugal following closely behind (Figure 4d).

Thirteen countries and territories had an ASPR >6000 per 100,000 in 2021. The highest ASPR was observed in Uganda (7769.97 per 100,000 [95% UI: 6262.35–9849.65]). Uganda, Greenland, Palestine, Angola, and Tunisia were among the top five in ASPR (Figure 4e). There were only seven countries and territories with an ASDR of more than 1000 per 100,000 persons in 2019. Since 2020, the number of countries and territories with an ASDR of more than 1000 per 100,000 persons has reached as high as 28 in 2021. Greenland had the highest level (1509.78 per 100,000, 95% UI: 999.39–2188.17). Greenland, Uganda, Palestine, Lesotho, and Tunisia had the five positions with the highest ASDRs (Figure 4f).

### 3.3. Regional and SDI Burdens of Depressive Disorder

**Figure 5:**
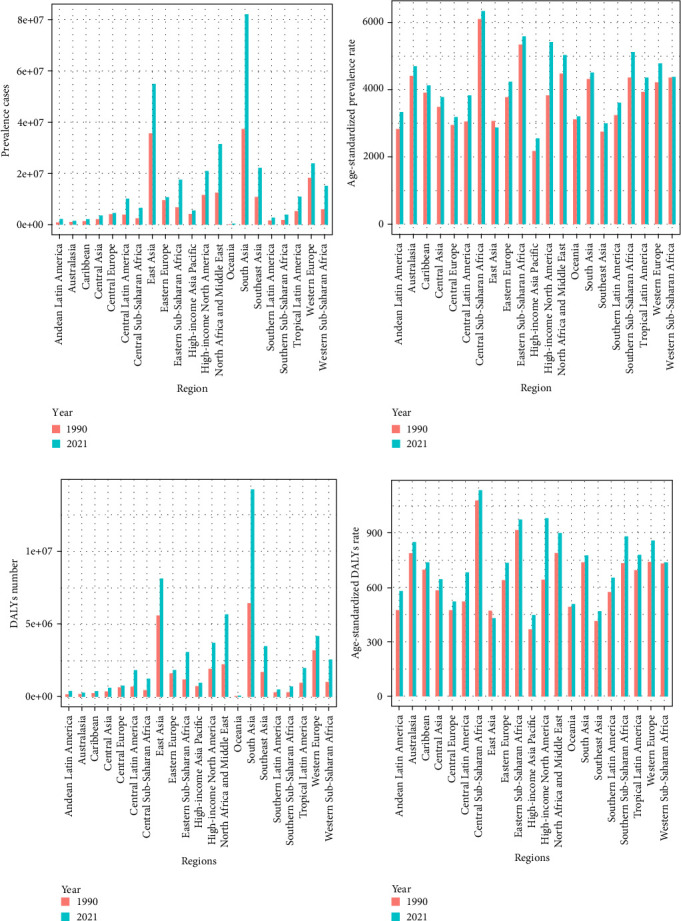
Prevalence cases, the DALYs, the ASPRs, and the ASDRs of depression in 21 regions. (a) Prevalence cases; (b) DALYs; (c) ASPRs; (d) ASDRs. ASDR, age-standardized DALY rate; ASPR, age-standardized rates of prevalence; DALY, disability-adjusted life-year.

South Asia had the highest number of cases in 1990 and 2021, with 37.19 million (95% UI: 33.19–42.42 million) in 1990 and 82.02 million (95% UI: 73.14–93.54 million) in 2021. There was an increase of 220.54% in the number of prevalent cases and 221.62% in the number of DALYs from 1990 to 2021. East Asia ranked second, with a slight difference in prevalent cases in 1990 but a significant gap in 2021, with 35.54 million (95% UI: 32.09–39.67 million) in 1990 and 54.87 million (95% UI: 48.92–61.34 million) in 2021. There was an increase of 154.39% for prevalent cases and an increase of 145.42% for DALYs from 1990 to 2021 (Table 1 and Figures 5a,c).

Central sub-Saharan Africa had the highest ASPR (6100.87 per 100,000 [95% UI: 5269.08–7235.87]) in 1990 and (6337.03 per 100,000 [95% UI: 5236.44–7669.98]) in 2021. Eastern sub-Saharan Africa had the second highest ASPR (5330.37 per 100,000 [95% UI: 4758.72–6057.56]) in 1990 and (5576.42 per 100,000 [95% UI: 4939.64–6372.72]) in 2021. They also shared the two highest ASDRs in 1990. In 2021, the sorting changed. Central sub-Saharan Africa remained the leader in ASDR, and the runner-up was high-income North America, with an increase of 153.39% in ASDR from 1990 to 2021 (Table 1 and Figures 5b,d).

**Figure 6:**
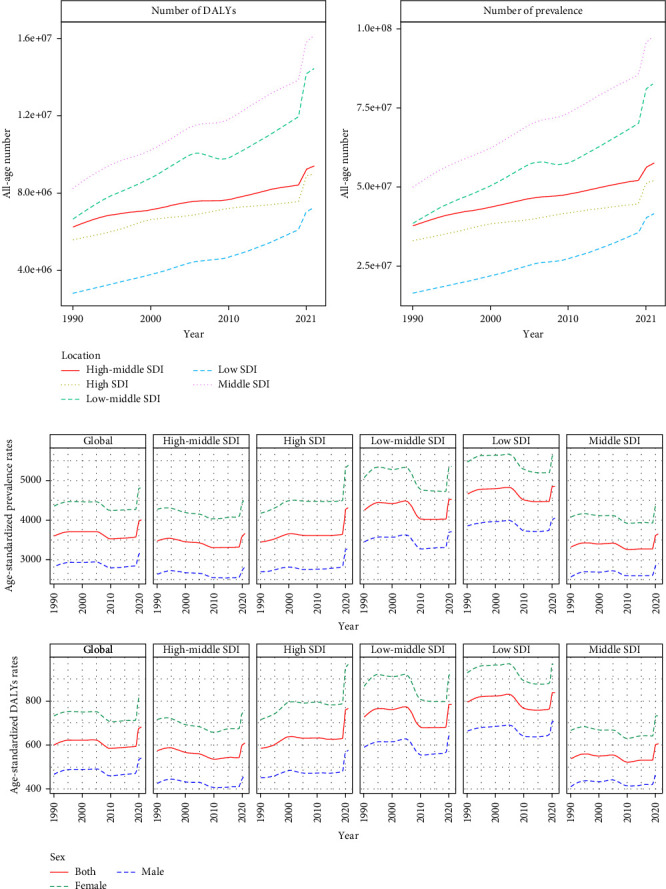
Tendencies in prevalence and DALYs of depression, 1990–2021. (a) Prevalent cases and DALYs; (b) age-standardized prevalence rate (ASPR) and age-standardized disability-adjusted life-year (ASDR). DALY, disability-adjusted life-year; SDI, sociodemographic index.

In addition, regional disparities in the burden of depression are significant. Prevalent cases and DALYs between SDI regions showed some differences in the past 32 years, with low-SDI regions having the lowest prevalence of cases and DALYs and middle-SDI regions having the highest prevalence of cases and DALYs. Lower growth rates were observed in the high-middle- and high-SDI regions, whereas more rapid growth rates were experienced in the middle- and low-middle-SDI regions. The fastest growth, which increased by more than two and a half in 32 years, appeared in low-SDI regions (Figure 6a).

For the tendencies of ASPR and ASDR, the global ASPR and ASDR slightly declined from 1990 to 2019 but significantly increased in 2020 and 2021. The lowest rates were noted in the middle-SDI region, and the highest rates were noted in the low-SDI region. These two rates decreased in most regions from 1990 to 2019, with a noticeable change of one turning point occurring in the low-middle and low-SDI regions in 2005, leading to a significant decreasing trend from 2005 to 2010. An exception to this trend was observed in high-SDI regions, where an increasing trend appeared from 1990 to 2019; enormous increase in ASPR and ASDR appeared in all regions from 2020 to 2021, whereas high-SDI regions contributed the largest increase in growth. Additionally, the ASPR and ASDR were significantly greater in women than in men (Figure 6b).

### 3.4. Associations of the SDI With the ASPR and ASDR

**Figure 7:**
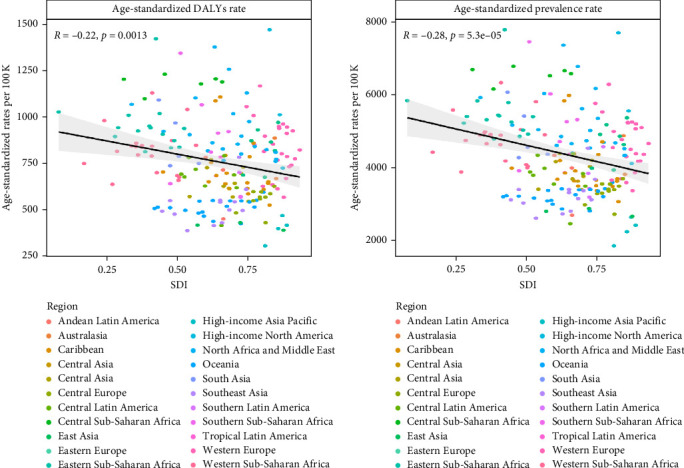
Associations of the SDI with the ASDR and ASPR, 2021. (a) SDI and the ASDR; (b) SDI and the ASPR. The points represent countries or territories. The solid line color black represents the expected values across the SDIs; the shadow represents the 95% confidence intervals; the Pearson correlation coefficients and *p* values are denoted. ASDR, age-standardized DALY rate; ASPR, age-standardized rates of prevalence; SDI, sociodemographic index.

Figure 7 shows the associations of ASPR and ASDR with the SDI in 2021. An evident decreasing trend and a statistically significant association between the ASPR and the SDI (*R* = −0.22, *p*=0.0013), as well as between the ASDR and the SDI (*R* = −0.28, *p* < 0.001), were observed.

## 4. Discussion

Depressive disorder is a primary public health challenge that affects individuals of all ages. It is linked to negative health consequences and decreased life expectancy [18]. This study examines the global burden of depression via the latest GBD data, emphasizing the difference in spatiotemporal trends in depression burden between 1990–2019 and 2019–2021. In addition to exploring the complex impact of COVID-19 on the burden of depression across regions and groups, we also discuss the sudden decline in the disease burden from 2005 to 2010, which has been overlooked in previous studies. We found that a vibrant social economy, better social integration policies and measures, a high degree of cooperation between the public and the government, and a fair and harmonious social atmosphere are conducive to managing and fighting depression. The findings of this study offer valuable insights for regional governments in developing pertinent policies and treatment strategies for depression.

Our study revealed a greater than 1.8-fold increase in the prevalence and DALYs of global depressive disorder from 1990 to 2021. This finding suggested that the immense economic burden caused by depressive disorder may have deteriorated in recent decades, which aligns with the findings from a 2019 study [19]. This is likely linked to the growing social pressure resulting from uneven social development, population growth and aging [20], and major outbreaks of epidemics such as coronavirus disease 2019 (COVID-19) [21]. Notably, advancements in depression screening tools have made obtaining more detailed and precise clear data easier for medical and government-related departments [22, 23]. However, the increase in disease burden was not consistent across regions, age groups, or sexes [10]. From 1990 to 2019, the ASRs slightly declined, with a 1.32% decrease in the ASPR and a 1.84% decrease in the ASDR. This trend may be attributed to increasing economic development and living standards, leading to a greater demand for medical services, particularly mental health services. People are placing more importance on mental health and seeking more mental health services, resulting in a reduction in years of life lost due to improved global diagnosis and treatment of the disease.

A prominent decline occurred from 2005 to 2010 (4.86% decrease in the ASPR and 6.09% decrease in the ASDR), with the majority of the contributions occurring in the low-middle- and low-SDI regions. As shown in Figure 7, there is an evident decreasing trend between the ASPR and the SDI, as well as between the ASDR and the SDI. Perhaps we can obtain some explanations for the turning point of the trend in 2005 through analyzing the SDI data worldwide from 1990 to 2021.

Since 1990, the SDI values of all 204 countries and territories have increased to varying degrees; however, the SDI rankings of most countries have been relatively stable. The ranking changes of a few countries occurred mainly in low-middle- and low-SDI regions and from 2005 to 2010. In 2005, 54 countries (South Africa, China, Thailand, Turkey, Cuba, Brazil, etc.) belonged to the low-middle-SDI region (SDI: 0.45–0.61), and 51 countries (Pakistan, India, Sudan, etc.) belonged to the low-SDI region (SDI: 0–0.45). In 2010, these populous developing countries made remarkable progress. South Africa, China, Thailand, Turkey, and Cuba entered the middle-SDI region (SDI: 0.61–0.69), and India met the low-middle-SDI standard. China experienced the most significant increase in ranking from 2005 to 2010 (SDI values increase by an average of 0.011 per year; 11 places forward, from 102nd in 2005 to 91st in 2010). Sudan was second (5 places forward, from the 160th in 2005 to the 155th in 2010), although it was still in the low-SDI region (GBD 2019, SDI 1950–2019).

According to public reports, from 2005 to 2010, China's economic growth rate reached its smooth and fast stage in history, and its GDP rose from the fifth largest to the second largest in the world. According to the 2011 Chinese National Government Work Report, medical and health expenditures have increased annually over the past 5 years. The year 2005 was a time of change in China's way of providing mental health services. Through the “686 Project," it gradually transformed from hospital-centered to strengthening community services [24]. In 2005, the project established 60 demonstration sites in 30 provinces, covering ~1/30 of the country's population. On the basis of China's large population size and heavy disease burden, we believe that China's enormous investment in controlling mental illness from 2005 to 2010 was a key factor in the amazing turning point of the depression burden in the low–middle-SDI region in 2005. Similarly, with good economic development from 2005 to 2010, some countries in low-SDI areas, such as India and Sudan, also made their own contributions to reducing the regional disease burden. The tremendous efforts made by these developing countries to control the disease burden have had a positive impact on the burden of depression worldwide.

There was another significant turning point in the trends of ASPR and ASDR in 2019. With the outbreak of COVID-19, the global ASPR and ASDR for depression experienced an astonishing jump from 2019 to 2021, resulting in an increase of nearly 11% in the ASPR and an increase of nearly 13% in the ASDR. COVID-19 has influenced all areas of society, including economic markets, industry and agriculture, healthcare, etc. [21]. The WHO 2020 speculated that further measures such as quarantine affect people's daily activities and livelihoods and may lead to more mental problems such as insomnia, anxiety, and depression; increases in substance use disorders; and self-harm or suicide. Millions of people are unemployed and struggle for food, shelter, and livelihoods. Therefore, many uncertainties lead to depression, suicide, and self-harm [21]. The COVID-19 virus is effective at entering the central nervous system [25–27]. “Postviral syndrome is a mixture of organic and psychiatric disease, and definitive confirmation of a viral etiology will not reduce the psychiatric symptoms of those affected” [28]. After the previous major epidemic, approximately one quarter of all survivors of deadly infection experienced symptoms of anxiety, depression, and severe cognitive problems [29, 30]. Compared with 1990–2019, the unexpected COVID-19 outbreak increased the global prevalence of DALYs, ASPR, and ASDR for mental disorders, including depression, from 2019 to 2021 and changed the trend of disease burden. Among the 21 GBD regions, the only exceptions to the decline in EAPC for ASPR and ASDP from 1990 to 2021 occurred in East Asia. This might be attributed to the timely implementation of epidemic prevention and strict control measures by the government and the high level of cooperation from the public.

We reported that women had a greater prevalence of depression and higher DALYs than males did, findings that are consistent with those of a previous study [15]. There are many reasons for this: females are more likely to encounter adverse events that affect their mood, such as bullying, postpartum depression, and sexual abuse, which might lead to an increased prevalence of depression [31–33]; self-repression and attention to social comparison information are more closely related to psychopathology for women than for men [34]. Notably, the ASPR and ASDR of depression decreased in females but increased in males from 1990 to 2019. This subtle difference has not been mentioned in previous studies. This was partly because women around the world have become better positioned in recent decades, taking better care of themselves and adopting healthier lifestyle habits. Nevertheless, the ASPR and ASDR of depression in females grew faster from 2019 to 2021. Although the COVID-19 pandemic has made public health security factors prominent, other factors related to unfairness have not disappeared. During the outbreak of the virus, females still had to become pregnant and continue nurturing [35–38]. Quarantine and lockdown measures led to an increase in cases of inescapable domestic violence for women and children (WHO 2020). Therefore, the pandemic has had a serious impact on females, which might lead to more mental health issues, including depression.

The population aged 20–54 years accounted for the majority of the prevalent cases and DALYs from 1990 to 2021. Notably, there were mild declines in the percentages of prevalent cases and DALYs in those aged <20 and 20–54 years, and the percentage in those aged 55+ years showed sustained growth before 2019. From 2020 to 2021, owing to the increases in the percentages of those aged <20 and 20–54 years, the percentage of those aged 55+ years decreased relatively.

In elderly individuals, depression is the second most common psychiatric disorder [6]. A review of 48 studies revealed that the global prevalence of depression among elderly individuals was as high as 28.8% [39]. Health problems in later life, including chronic pain, Parkinson's disease, diabetes, heart disease, stroke, and other high-risk diseases, which can affect the mental state of elderly individuals, are highly correlated with geriatric depression [40–43]. Many older patients do not respond optimally to antidepressants [44]. In addition, changes in social roles after retirement, reduced social activities, and the continued loss of important friends might also worsen mood, which could have an impact on treatment outcomes [45]. The cost of health care and informal care for depressed elderly people is several times greater than that for nondepressed elderly people [46–48]. With population growth and aging in many countries, geriatric depression is becoming a serious challenge. Although the proportion of depression in the elderly population slightly decreased from 2020 to 2021 due to the COVID-19 outbreak, preventing and controlling depression in the elderly population should still be a priority.

According to a published 2020 technical report, COVID-19-induced shocks and uncertainty had considerable impacts on personal networking, the job market, and the world economy. Although isolation helps control the risk of infection, being separated from family, friends, and other social support systems could lead to mental problems, such as adult anxiety and depression [49], as well as child restlessness and violence [21]. The COVID-19 pandemic disrupted the normal order of life, raising concerns about its impact on youth mental health [50]. In addition, in the past few years of pandemics, many students have encountered difficulties in participating in team sports and have not met the recommended PA levels, which are associated with mental health outcomes for high school students [51]. In this age of social media, people were surrounded by rumors and misinformation about the pandemic, causing widespread fear, anxiety, and stress [21], which had a greater impact on adults and teenagers with greater social needs. To alleviate these issues, the WHO issued several guidelines to protect the mental health of people, with a special focus on children and women.

There were increasing trends in the APC for actual values from 1990 to 2005, but some small fluctuations with relatively flat trends in ASRs occurred from 1994 to 2005. One platform period subsequently appeared in the APC for actual values from 2005 to 2010, and interestingly, decreasing trends in ASPR and ASDR were observed during the same period. These findings indicate that the profiles of depression improved to some extent globally from 2005 to 2010. Unfortunately, the trend of APC for actual values has rapidly increased, and the relevant trends of ASPR and ASDR have shown increasing trends since 2010, with a particular increase from 2019 to 2021. Overall, although increasing trends in prevalent cases and DALYs for actual values have been observed over the past decades, the global prevalence and DALY decreased from 1990 to 2019 but increased from 2020 to 2021, which might be due to the comprehensive effects of increasing social pressure, improvements in disease cognition and intervention, and the COVID-19 outbreak worldwide.

We found that India had the highest prevalence of cases and a leading DALY, with China and the USA following closely. This could be attributed to their large populations. In our study, the highest ASPR was observed in Uganda in 2021. Notably, Greenland became the country with the highest prevalence rate and DALY and ASDR rates worldwide in 2021.

Greenland has experienced major social and political transformation in recent decades. The transition from a traditional economy based on hunting and fishing in small settlements to a modern economy based on urbanization and wage labor has caused changes in lifestyles, life values, and family structure with both positive and negative health consequences [52–54]. In terms of high socioeconomic inequality [54] and negative family relationships [55], the consumption of alcohol and cannabis and sexual risk behavior are greater than those in other European countries. A study from 1999 suggested that anxiety and depression were correlated with unemployment, sexual abuse, violence, and suicide among relatives or friends for those who lived in Greenland [56]. Since 1960, Greenland has one of the regions with the highest number of suicides in the world. Research on psychiatric disorders in Greenland has focused mostly on the impact of social change on health and disease. Despite differences in sex and age, most studies have identified known socioeconomic risk factors [57]. The studies cited above further examined the impact of cultural factors on mental health and revealed that better-integrated groups in modern Greenlandic society had better mental health, whereas a significant group appeared to be less socially integrated and more mentally ill [56]. Berry's theory of acculturate stress in the context of cultural change [58, 59] also suggested that cultural integration produced the best psychosocial outcomes, whereas marginalization was associated with psychopathology [60]. It seems that better social integration policies and measures might help alleviate severe levels of depression in Greenland.

The reported findings indicate that Uganda has a relatively high population density and a high prevalence of tropical diseases, which might be related to Uganda having the highest ASPR for depression in the world. Uganda had one of the highest rates of HIV infection. Although the peak of the epidemic had passed and new cases had decreased, the number of people affected by the virus remained at a high level. HIV patients face discrimination, often leading to negative emotions and even depression [61]. This finding might suggest that controlling HIV outbreaks could have a positive effect on depression. In addition, the outbreak of COVID-19 further worsened the disease burden of depression in the past few years.

The largest increases in prevalence and DALYs were identified in South Asia and East Asia. These increases were particularly notable in the middle- and low-middle-SDI regions. This trend could be attributed to the significant improvements in population size, economic conditions, and education levels over the past 32 years in these regions. The escalating social pressures faced by residents might also contribute to the increasing prevalence of depression. As the social economy advances, individuals experience heightened social pressures. Research has established a strong correlation between educational attainment and cognitive function. Educational level not only impacts an individual's susceptibility to depression but also influences their spouses and parents [62].

In 1990, Central sub-Saharan Africa and Eastern sub-Saharan Africa had the two highest ASPRs and ASDRs. In 2021, high-income North America surpassed Eastern sub-Saharan Africa and occupied second place in ASDR, with a slight advantage. Compared with other regions, high-income North America, which has high economic income and high SDI, has experienced greater growth in ASPR and ASDR, which aligns with our previous observations. The findings of some studies have suggested that in the context of the impact caused by COVID-19, the largest drop in earnings and employment has occurred in poor household areas, whereas the greatest reduction in spending, such as entertainment and transport, has been recorded in rich areas [63].

Further analysis revealed a continued increasing trend in depression across all five SDI regions. Both the greatest prevalence and DALYs and accelerated growth were observed in the middle-SDI and low-middle-SDI regions. Following age standardization, the low-SDI and high-SDI regions presented the highest ASPR and ASDR. Moreover, considerable decreasing trends were noted in most regions from 1990–2019, which had a positive impact on the global temporal trend of depression. Significant increases were observed in the five SDI regions in 2020 and 2021, with the largest increase occurring in the high-SDI region. In general, depression was more prevalent in high-income and high-SDI regions, whereas the burden was notable in low-income and low-SDI regions [15]. However, through visual analysis of the data, we also noted that from 2019 to 2021, the spread of the COVID-19 epidemic brought unexpected challenges to high-income and high-SDI regions. The burden of depression has increased the fastest in these regions, which has become one of the regions with the heaviest depression burdens.

The global burden of depression presents diverse situations in various regions for several reasons, including economic, medical and educational imbalances, and limited disease diagnosis capabilities. Additionally, differences in the level of attention given by governments in different regions to the disease play a significant role [62, 64, 65]. Furthermore, social and human factors such as religious beliefs and cultural customs also impact the disease burden in different regions.

Although significant progress has been made in elucidating the pathophysiology of depression and treating depressive symptoms, the pathogenesis of this disease remains unclear, and current treatments remain ineffective for many patients [66]. This poses a challenge for effectively preventing and controlling depression. Given the high prevalence and heavy burden of depression, further research is needed to address this issue [67].

The disease burden is uneven across countries and regions and has increased to varying degrees in recent years. Countries should adopt feasible intervention measures to change or slow the rising trend, including improving people's awareness of the disease, conducting psychological interventions, improving facilities, etc., and call for relevant reform systems to focus on vulnerable groups and better improve mental health.

Our study provided some unique analyses of the burden of depression by employing DALY, ASPR, ASDR, and EAPC. However, this study still has limitations: (I) Although the data in the GBD study were standardized to the greatest extent possible, the inconsistent data quality across different countries and regions or even the lack of original data might introduce various biases. (II) A detailed analysis of potential disease-related risk factors is lacking. (III) The differences between dysthymia and MDD have neither been addressed, nor have related comorbidities been addressed. We anticipate that the GBD database will provide more comprehensive disease-related risk factors through continuous updates and further differentiate these disorders more scientifically to facilitate more thorough analysis [68]. (IV) Subnational disease burden analysis is lacking. To obtain a more detailed picture of the burden of depression, subnational data need to be extracted and analyzed in the future to assess subnational differences in disease burden and provide a reference for subnational health policies.

## 5. Conclusion

The global burden of depression has remained high and has worsened since the pandemic. The governments of all countries should actively develop a balanced social economy, help disadvantaged groups improve people's livelihood, and create a harmonious society. Notably, in major public events, public mental health should be included in the priority program.

## Figures and Tables

**Table 1:** Prevalence, DALY and the corresponding ASRs in 1990 and 2021 and the ASR-related EAPCs from 1990 to 2021.

Location	Prevalence				DALY				EAPC (1990–2021)	
	No. × 10^5^ (95% UI) in 1990	Age-standardized no. × 10^−5^ in 1990	No. × 10^5^ (95% UI) in 2021	Age-standardized no. × 10^−5^ in 2021	No. × 10^5^ (95% UI) in 1990	Age-standardized no. × 10^−5^ in 1990	No. × 10^5^ (95% UI) in 2021	Age-standardized no. × 10^−5^ in 2021	ASPR no. (95%Cl)	ASDR no. (95%Cl)
Global	1763.27 (1589.31–1993.75)	3599.67 (3251.91–4023.21)	3324.10 (2977.42–3761.02)	4006.82 (3581.26–4539.01)	296.75 (207.49–402.06)	600.52 (420.94–818.45)	563.30 (393.40–765.38)	681.14 (475.19–923.83)	0.11 (0.09–0.14)	0.13 (0.11–0.16)
Sex										
Female	1076.75 (970.04–1215.55)	4358.15 (3938.97–4883.83)	2012.6 (1798.7–2284.5)	4822.12 (4316.38–5483.35)	182.10 (127.39–246.59)	731.70 (512.35–996.70)	341.19 (237.95–462.20)	821.17 (570.96–1110.43)	0.11 (0.08–0.13)	0.12 (0.10–0.15)
Male	686.52 (618.18–773.34)	2833.00 (2551.62–3163.66)	1311.4 (1174.0–1478.8)	3186.4 (2853.4–3604.3)	114.65 (80.09–156.06)	468.06 (326.20–640.13)	222.11 (154.98–303.15)	540.51 (377.31–735.48)	0.12 (0.10–0.15)	0.15 (0.13–0.18)
SDI										
High	332.10 (301.91–367.98)	3446.86 (3133.42–3829.18)	520.33 (468.19–579.86)	4312.34 (3857.18–4866.01)	56.00 (39.05–76.24)	584.72 (407.23–792.38)	90.08 (62.64–121.10)	766.07 (535.99–1026.59)	0.25 (0.21–0.30)	0.31 (0.26–0.37)
High-middle	377.33 (341.85–420.77)	3478.40 (3151.80–3862.92)	576.26 (513.57–641.98)	3666.90 (3280.98–4119.10)	62.63 (43.82–85.12)	575.27 (404.6–784.67)	94.08 (66.32–128.20)	610.08 (424.53–831.09)	0.05 (0.02–0.09)	0.06 (0.02–0.10)
Middle	500.84 (451.56–562.49)	3321.97 (3002.47–3712.10)	979.74 (877.85–1096.01)	3654.36 (3272.50–4075.44)	82.70 (57.29–112.19)	537.88 (375.23–735.24)	161.58 (113.08–219.65)	605.91 (424.52–824.66)	0.10 (0.08–0.12)	0.13 (0.10–0.15)
Low-middle	386.07 (343.34–443.37)	4250.24 (3815.69–4807.30)	828.61 (736.00–954.85)	4528.90 (4043.41–5176.25)	66.74 (45.80–90.94)	726.74 (502.50–981.12)	144.40 (99.62–194.50)	784.07 (542.47–1059.21)	0.07 (0.03–0.11)	0.08 (0.04–0.13)
Low	165.33 (145.21–192.77)	4664.77 (4155.86–5346.20)	416.65 (363.24–485.29)	4849.64 (4260.97–5554.66)	28.41 (19.43–38.94)	795.50 (545.59–1072.90)	72.73 (49.36–99.61)	837.53 (569.85–1140.08)	0.04 (0.00–0.07)	0.05 (0.01–0.09)
Region										
Andean Latin America	8.67 (7.53–10.07)	2821.69 (2476.53–3232.06)	22.25 (19.06–26.03)	3325.81 (2851.30–3880.45)	1.47 (1.00–2.01)	474.69 (323.60–644.82)	3.88 (2.61–5.42)	578.74 (389.32–804.97)	0.18 (0.07–0.31)	0.22 (0.08–0.38)
Australasia	9.55 (8.49–10.81)	4407.64 (3912.81–4998.58)	15.43 (12.74–18.44)	4691.88 (3904.49–5719.89)	1.70 (1.18–2.30)	787.74 (546.91–1065.63)	2.74 (1.86–3.85)	849.31 (569.13–1201.644)	0.06 (−0.09−0.24)	0.08 (−0.10−0.28)
Caribbean	12.63 (11.02–14.62)	3911.57 (3420.76–4461.46)	20.01 (17.81–24.64)	4121.50 (3512.09–4870.28)	2.27 (1.55–3.08)	697.36 (470.04–954.74)	3.74 (2.58–5.20)	737.81 (507.64–1028.88)	0.05 (−0.03−0.15)	0.06 (−0.04−0.16)
Central Asia	20.50 (17.97–23.39)	3482.87 (3058.01–3966.88)	35.71 (30.79–41.49)	3773.71 (3243.38–4386.79)	3.45 (2.36–4.70)	582.56 (401.13–795.95)	6.09 (4.13–8.42)	644.28 (441.83–883.56)	0.08 (0.01–0.16)	0.11 (0.02–0.20)
Central Europe	40.36 (35.94–45.66)	2946.11 (2624.38–3340.06)	45.90 (40.47–52.15)	3171.90 (2789.03–3611.52)	6.46 (4.50–8.74)	472.39 (328.24–642.45)	7.45 (5.16–10.06)	521.75 (358.80–710.10)	0.08 (0.03–0.13)	0.10 (0.05–0.17)
Central Latin America	39.55 (34.99–45.24)	3046.79 (2703.86–3461.76)	101.46 (90.08–116.09)	3825.60 (3399.91–4375.35)	6.86 (4.70–9.35)	522.11 (361.21–711.52)	18.13 (12.43–24.86)	682.94 (468.28–935.02)	0.26 (0.21–0.31)	0.31 (0.25–0.38)
Central Sub-Saharan Africa	24.14 (20.50–29.09)	6100.87 (5269.07–7235.87)	66.66 (54.47–82.83)	6337.03 (5236.44–7669.98)	4.33 (2.89–5.97)	1080.06 (735.71–1470.36)	12.14 (8.03–16.87)	1136.91 (756.88–1588.38)	0.04 (−0.07−0.17)	0.05 (−0.07−0.19)
East Asia	355.45 (320.94–396.75)	3059.66 (2765.46–3392.61)	548.69 (489.18–613.36)	2870.61 (2583.73–3205.35)	55.89 (38.63–75.45)	470.81 (329.33–635.95)	81.20 (57.49–110.46)	429.67 (304.25–585.42)	−0.06 (−0.10-−0.03)	−0.09 (−0.13-−0.05)
Eastern Europe	95.37 (85.29–107.30)	3774.06 (3369.08–4255.23)	106.19 (93.94–118.73)	4231.75 (3729.90–4771.11)	16.09 (11.07–21.96)	638.26 (441.05–874.64)	18.18 (12.73–24.98)	735.83 (510.58–1005.60)	0.12 (0.07–0.19)	0.15 (0.09–0.23)
Eastern Sub-Saharan Africa	67.24 (58.95–78.44)	5330.37 (4758.72–6057.56)	175.13 (152.28–204.93)	5576.42 (4939.64–6372.72)	11.61 (7.98–15.83)	917.79 (628.42–1236.02)	30.81 (20.81–41.97)	974.65 (668.67–1308.67)	0.05 (0.00–0.10)	0.06 (0.01–0.12)
High-income Asia Pacific	41.45 (37.60–45.83)	2168.62 (1975.10–2399.34)	54.62 (48.82–60.75)	2545.16 (2266.73–2892.38)	7.01 (4.83–9.55)	367.93 (252.40–504.46)	9.30 (6.46–12.48)	447.86 (307.99–606.65)	0.17 (0.11–0.24)	0.22 (0.14–0.30)
High-income North America	115.48 (104.36–127.03)	3817.10 (3439.49–4244.85	207.87 (188.19–231.40)	5408.26 (4846.90–6049.72)	19.26 (13.42–26.21)	640.75 (446.86–864.81)	37.02 (25.81–49.38)	982.78 (685.33–1322.41)	0.42 (0.34–0.49)	0.53 (0.46–0.61)
North Africa and Middle East	124.48 (107.95–147.29)	4468.97 (3904.16–5216.63)	313.78 (269.47–369.11)	5024.69 (4346.40–5857.38)	22.31 (15.07–30.84)	788.13 (536.47–1078.26)	56.59 (37.33–77.76)	900.68 (598.46–1242.73)	0.12 (0.07–0.18)	0.14 (0.08–0.21)
Oceania	1.63 (1.41–1.93)	3116.26 (2709.63–3653.81)	3.90 (3.21–4.73)	3201.99 (2654.70–3811.00)	0.27 (0.18–0.37)	490.41 (337.66–672.60)	0.63 (0.41–0.89)	507.90 (329.20–704.04)	0.03 (−0.07−0.14)	0.04 (−0.09−0.19)
South Asia	371.92 (331.93–424.17)	4307.02 (3862.34–4834.86)	820.18 (731.45–935.36)	4500.48 (4034.93–5106.63)	64.30 (44.23–87.69)	737.68 (508.98–996.63)	142.48 (98.69–192.89)	777.80 (542.56–1049.65)	0.04 (0.01–0.09)	0.05 (0.01–0.10)
Southeast Asia	107.42 (94.59–121.91)	2742.55 (2435.89–3111.36)	221.12 (195.31–250.68)	2991.56 (2647.86–3401.76)	16.66 (11.52–22.57)	413.36 (286.16–564.46)	34.49 (23.84–47.08)	467.63 (323.74–634.88)	0.09 (0.06–0.13)	0.13 (0.09–0.18)
Southern Latin America	15.76 (14.00–17.94)	3231.93 (2879.16–3675.04)	26.19 (22.26–30.72)	3605.13 (3048.18–4246.28	2.81 (1.95–3.84)	575.29 (398.25–784.19)	4.70 (3.16–6.51)	652.62 (439.62–904.82)	0.12 (−0.02−0.25)	0.13 (−0.02−0.29)
Southern Sub-Saharan Africa	17.91 (16.05–20.32)	4352.90 (3926.75–4875.22)	39.58 (35.10–45.39)	5113.03 (4540.53–5818.20)	3.04 (2.11–4.12)	733.16 (511.87–997.02)	6.85 (4.71–9.47)	880.69 (609.09–1219.63)	0.17 (0.11–0.25)	0.20 (0.13–0.29)
Tropical Latin America	52.47 (47.15–59.47)	3931.87 (3542.56–4398.76)	109.46 (97.14–124.22)	4352.09 (3871.00–4948.92)	9.36 (6.51–12.77)	694.30 (485.38–940.87)	19.57 (13.54–26.57)	780.15 (539.06–1062.15)	0.11 (0.05–0.17)	0.12 (0.06–0.20)
Western Europe	182.67 (166.60–201.20)	4212.39 (3827.55–4693.04)	237.87 (211.37–267.12)	4778.95 (4207.89–5528.96)	31.77 (22.44–42.96)	739.93 (517.88–999.75)	41.69 (29.52–56.10)	858.16 (600.37–1164.62)	0.13 (0.07–0.21)	0.16 (0.09–0.25)
Western Sub-Saharan Africa	58.63 (51.88–67.44)	4364.98 (3895.29–4942.00)	151.23 (132.70–175.50)	4372.19 (3880.29–4963.05)	9.88 (6.79–13.48)	732.24 (502.89–999.64)	25.60 (17.64–35.11)	736.53 (502.61–1002.77)	0.00 (−0.13−0.03)	0.01 (−0.03−0.05)

Abbreviations: DALYs, disability-adjusted life-years; UI, uncertainty interval.

## Data Availability

The datasets used and/or analyzed during the current study are available from the Global Burden of Disease (GBD) Data Exchange database (http://ghdx.healthdata.org/gbd-results-tool).
